# Integrated comparative transcriptome and physiological analysis reveals the metabolic responses underlying genotype variations in NH_4_^+^ tolerance

**DOI:** 10.3389/fpls.2023.1286174

**Published:** 2023-12-13

**Authors:** Haifei Chen, Wei Lv, Wenqi Zhang, Jie Zhao, Quan Zhang, Zhenhua Zhang

**Affiliations:** ^1^ College of Resources, Hunan Agricultural University, Changsha, China; ^2^ Key Laboratory of Agro-ecological Processes in Subtropical Region, Institute of Subtropical Agriculture, Chinese Academy of Sciences, Changsha, China

**Keywords:** nitrogen, NH_4_^+^ toxicity, NH_4_^+^ assimilation, phenylpropanoid biosynthesis, transcriptome analysis

## Abstract

Several mechanisms have been proposed to explain NH_4_^+^ toxicity. However, the core information about the biochemical regulation of plants in response to NH_4_^+^ toxicity is still lacking. In this study, the tissue NH_4_^+^ concentration is an important factor contributing to variations in plant growth even under nitrate nutrition and NH_4_^+^ tolerance under ammonium nutrition. Furthermore, NH_4_^+^ led to the reprogramming of the transcriptional profile, as genes related to trehalose-6-phosphate and zeatin biosynthesis were downregulated, whereas genes related to nitrogen metabolism, camalexin, stilbenoid and phenylpropanoid biosynthesis were upregulated. Further analysis revealed that a large number of genes, which enriched in phenylpropanoid and stilbenoid biosynthesis, were uniquely upregulated in the NH_4_^+^- tolerant ecotype Or-1. These results suggested that the NH_4_^+^-tolerant ecotype showed a more intense response to NH_4_^+^ by activating defense processes and pathways. Importantly, the tolerant ecotype had a higher ^15^NH_4_^+^ uptake and nitrogen utilization efficiency, but lower NH_4_^+^, indicating the tolerant ecotype maintained a low NH_4_^+^ level, mainly by promoting NH_4_^+^ assimilation rather than inhibiting NH_4_^+^ uptake. The carbon and nitrogen metabolism analysis revealed that the tolerant ecotype had a stronger carbon skeleton production capacity with higher levels of hexokinase, pyruvate kinase, and glutamate dehydrogenase activity to assimilate free NH_4_^+^, Taken together, the results revealed the core mechanisms utilized by plants in response to NH_4_^+^, which are consequently of ecological and agricultural importance.

## Introduction

Nitrogen (N) is an essential plant macronutrient that is required for growth and is thus important in agricultural production. NH_4_^+^-based fertilizers contain commonly used N forms synthesized via the Haber–Bosch process (Halvorson et al., 2014). While NH_4_^+^ is the preferred form of N for plants at low concentrations, it is toxic to plants at high concentrations (von Wirén et al., 2000; Britto and Kronzucker, 2002). As NH_4_^+^ escapes little with water in soil and elevated CO_2_ reduces nitrate reductions in C3 species, a “more NH_4_^+^ solution” was considered to mitigate nitrogen pollution and improve crop yields (Rubio-Asensio and Bloom, 2016; Subbarao and Searchinger, 2021). However, excess NH_4_^+^/NH_3_ atmospheric depositions have attracted attention in recent decades as they caused environmental problems related to species richness and composition (Stevens et al., 2004; Clark and Tilman, 2008; Duprè et al., 2010). The mechanisms underlying NH_4_^+^ toxicity in plants are consequently of ecological and agricultural importance.

NH_4_^+^ toxicity causes a retardation in plant growth, shortened roots, reduced root gravitropism, and leaf chlorosis (Britto and Kronzucker, 2002; Li et al., 2014). Several mechanisms have been proposed to explain NH_4_^+^ toxicity, including the depletion of organic acids (Hachiya et al., 2010), deficiency of cations (Hoopen et al., 2010), futile transmembrane NH_4_^+^ cycling (Britto et al., 2001; Li et al., 2022), photodamage to photosystem II (Dai et al., 2014), reactive oxygen and nitrogen species (RONS) induced oxidative stress (Liu et al., 2022), and the disruption of hormonal homeostasis (Li et al., 2012). The reactions of NH_4_^+^ conjugation to glutamic acid and the synthesis of glutamic acid are critical for the detoxification of NH_4_^+^, and are catalyzed by glutamine synthase (GS), glutamate synthase (GOGAT), and glutamate dehydrogenase (GDH) (Masclaux-Daubresse et al., 2006). Studies have shown that GS activity is upregulated by high external NH_4_^+^ levels and that NH_4_^+^-tolerant plants have higher GS activity and lower levels of NH_4_^+^ accumulation in their tissues (Cruz et al., 2006; Guan et al., 2016). In addition, NH_4_^+^ uptake is tightly controlled through the NH_4_^+^-dependent inhibition of AMT1 by the phosphorylation of Thr-460 (Straub et al., 2017).

In recent years, numerous genetic loci controlling NH_4_^+^ toxicity have been identified by screening for mutants. The Arabidopsis mutant *hsn1-1* and its alelic mutant *vtc1* are hypersensitive to NH_4_^+^ because of point mutations in the gene encoding GDP-mannose pyrophosphorylase (GMPase). Defects in protein N-glycosylation of *hsn1-1* have been linked to the root hypersensitivity phenotype (Qin et al., 2008). Based on leaf hypersensitivity to NH_4_^+^, *ammonium overly sensitive 1* (*amos1*) and *amos2* were identified. *amos1* was found to be an allelic mutation of EGY1 encoding a plastid metalloprotease. AMOS1/EGY1 mediated plastid retrograde signaling regulates NH_4_^+^-responsive genes to maintain chloroplast functionality (Li et al., 2012). An *ammonium tolerance mutant* (*amot1*) was identified as allelic to *EIN3*, which positively regulates ROS production and induces oxidative stress under NH_4_^+^ stress (Li et al., 2019a). Moreover, other genetic loci that control root development and gravitropism have been identified in Arabidopsis, including *auxin resistant 1* (*aux1*), *tiny root hair 1* (*trh1*), *dolichol phosphate mannose synthase1* (*dpms1*), and *gravitropism sensitive to ammonium 1* (*gsa1*) (Li et al., 2014).

Although the numerous genetic loci were found to associate with the toxicity of NH_4_^+^ nutrition, the function studies of individual genes cannot fully reflect the natural responses of plants to NH_4_^+^ toxicity. The tolerance of plants to NH_4_^+^ depends on the species and variety. For example, some rice cultivars have adapted to NH_4_^+^, while *Arabidopsis thaliana* and the *Brassicaceae* family are sensitive to NH_4_^+^ (Esteban et al., 2016; Li et al., 2021). In this study, we explore the cellular threshold of NH_4_^+^ concentration by investigated the *A. thaliana* natural accessions. More importantly, the core information about the biochemical regulation of plants in response to NH_4_^+^ toxicity was identified by comparative assessments of the sensitive and tolerant species.

## Materials and methods

### Plant material and culture conditions

A natural accessions of *Arabidopsis thaliana* were used in this study (**Table S1**). Surface-sterilized seeds were sown onto nutritional soil in a greenhouse (300 μmol photons m^-2^ s^-1^, 16 h photoperiod, 22°C) for 7 d. Then, eight uniformly growing plants of each ecotype were grown hydroponically with 4 L of nutritional media per pot, containing 1 mM Ca(NO_3_)_2_ or 1 mM (NH_4_)_2_SO_4_. The other essential nutrients in the two different nitrogen nutrient solutions were the same as our previous study (Chen et al., 2021), specially 1.25 mM KCl, 0.625 mM KH_2_PO_4_, 0.5 mM MgSO_4_, 25 μM Fe-EDTA, 17.5 μM H_3_BO_3_, 3.5 μM MnCl_2_, 0.25 μM ZnSO_4_, 0.05 μM NaMoO_4_, and 0.125 μM CuSO_4_. It is of note that the concentration of Ca^2+^ in the solution was uniformly set to 1.5 mM, and The pH was buffered to 6.0 using 2.5 mM MES. The *thaliana* natural accessions was grown under the NH_4_^+^ and NO_3_^-^.

The seedlings were first grown in 1 mM Ca(NO_3_)_2_ nutrient solution for 5 d and then transferred to nitrogen-free nutrient solution for 3 d. Subsequently, the *thaliana* natural accessions were grown under the NH_4_^+^ and NO_3_^-^ treatments for 8 days to investigate the NH_4_^+^ concentration and fresh weight. The culture solution was refreshed every 4 days.

### Measurement of the NH_4_^+^ and total nitrogen concentrations

NH_4_^+^ was extracted with deionized water using fresh rosette leaves. The supernatant was used to determine the NH_4_^+^ concentration after the samples were centrifuged at 12,000 × g. Briefly, 20 μL supernatant was mixed with 0.5 mL phenol solution (10 g/L Phenol, 100 mg/L sodium nitrosoferricyanide) and 5 mL sodium hypochlorite alkaline solution (Sodium hydroxide 5 g, sodium hydrogen phosphate 3.53 g, sodium phosphate 15.9 g, sodium hypochlorite solution (ω= 5.25%) 5 mL, dissolved in 500 ml deionized water). The NH_4_^+^ concentrations were measured colorimetrically using phenol hypochlorite (Berthelot reaction) colorimetry at 630 nm, and (NH_4_)_2_SO_4_ was used as a standard.

For total nitrogen (TN) measurement, the plants were sampled and then dried at 105°C for half an hour and further dried at 65°C until a constant weight. The samples were weighed 0.100g using a thousand balance, packed in 150 mL narrow-mouth triangular flasks, boiled with H_2_SO_4_-H_2_O_2_ until transparent and clear. The solution was transferred to a 50 mL volumetric flask and made up to volume. The solution was then filtered and analyzed using a continuous-flow analyzer AA3 (Autoanalyzer 3; SEAL, Germany). NUtE (Nitrogen utilization efficiency) was calculated as dry shoot biomass/shoot N.

### Enzyme activity

Fresh samples (100 mg) were homogenized in 3 mL of 50 mM Tris-HCl buffer (pH 8.0) containing 2 mM Mg^2+^, 2 mM DTT, and 0.4 M sucrose. After centrifuged at 10000 × *g* and 4°C for 10 min, the supernatant was then used to determine the activity of glutamine synthase. The activities of GS were measured according to the reference (Chen et al., 2018). Briefly, 0.5 mL supernatant was mixed with 1.6 mL reagent^①^, which is composed of 100 mM Tris-HCl (pH 7.4), 80 mM MgSO4, 20 mM glutamate, 20 mM cysteic acid, 2 mM EGTA and 80 mM hydroxyl-amine hydrochloride, and 0.7 mL reagent^②^, which is composed of 40 mM ATP. The 3 mL reaction mixture was incubated for 30 min at 37°C and was terminated by adding 1 mL FeCl_3_ reagent (88 mM FeCl_3_, 670 mM HCl and 200 mM TCA). After 10 min, the mixture was centrifuged at 4000 × *g* for 10 min, and the absorption value of supernatanta solution was determined at 540 nm wavelength.

NADH-dependent glutamate dehydrogenase was measured according to the reference (Groat and Vance, 1981). Briefly, fresh samples (100 mg) were homogenized in 3 mL of 50 mM Tris-HCl buffer (pH 8.0). 50 μL of the supernatant was mixed with the 950 μL reagent, which contained 100 μM NADH, 2.5 mM 2-oxoglutarate (2-OG), 200 mM NH_4_Cl. The enzyme activity was defined as the reduction of absorbance due to NADH at 340 nm. The assay of enzyme activity was performed according the instruction of commercial kit (NADH-GDH kit, BC1465, Solarbio).

Pyruvate (Pyr) concentrations were measured according to the reference with some modifications (Kachmar and Boyer, 1953). Pyr reacts with 2, 4-dinitrophenylhydrazine to form pyruvate-2, 4-dinitrophenylhydrazone, which could be determined by calorimetry. Briefly, 0.1 g Fresh samples (100 mg) were homogenized in 1 mL trichloroacetic acid (8%) and placed on ice for 3 min. After centrifuged at 8000 × *g* and 4°C for 10 min, 75 μL the supernatant was added to the enzyme label plate and added 25 μL 2, 4-dinitrophenylhydrazine (0.05%), to react 2 min. Finally, 125 μL 1.5 M NaOH was added, and the absorption value of the tube was determined at 520 nm wavelength. The assay was performed using the Micro Pyruvate Assay Kit (BC2205; Solarbio).

Pyruvate kinase (PK) activity was measured according to the reference (Lepper et al., 2010). The assay was performed according to the instructions of the Pyruvate Kinase Assay Kit (BC0540; Solarbio). Briefly, 0.1 g Fresh samples (100 mg) were homogenized in 3 mL extracting solution, which contained 100 mM Tris-HCl (pH 7.5), 10 mM β-mercaptoethanol, 12.5% (v/v) glycerine, l mM EDTA-Na_2_, 10 mM MgCl_2_, and 1% (m/v) PVP-40. After centrifuged at 8000 × *g* and 4°C for 10 min, 0.1 mL of the supernatant was mixed with 0.9 mL reagent, which contained 100 mM Tris-HCl (pH 7.5), 10 mM MgCl_2_, 0.16 mM NADH, 75 mM KCl, 5.0 mM ADP, 7.0 units L-lactate dehydrogenase (LDH), and 1.0 mM phosphoenolpyruvate (PEP). The absorption values at 340 nm wavelength were immediately recorded at 20s and 140s.

Hexose kinase (HXK) activity was measured according to the reference (Pancera et al., 2006). The assay was performed according to the instructions of the Hexose Kinase Assay Kit (BC0740; Solarbio). Briefly, 0.1 g Fresh samples (100 mg) were homogenized in 1 mL extracting solution. After centrifuged at 8000 × *g* and 4°C for 10 min, 10 μL the supernatant was added to the enzyme label plate, followed by 10 μL G-6-PDH solution (0.12 g/L) and 180 μL reagent, which 5 μl of a 2.0 mM NADP^+^ solution, 15 μl of a 0.1 M ATP solution, 50 μl of a 1 M glucose solution, and 110 μL of a 100 mM Tris–HCl buffer (pH 7.5). The absorption values at 340 nm wavelength were immediately recorded at 20s and 320s.

### ^15^N-NH_4_^+^ isotope tracing

The objective of ^15^N-labeling experiment after 3d of N starvation is investigate the transport capacity of both genotypes. Seedlings were first grown in 1 mM Ca(NO_3_)_2_ nutrient solution for 5 d, and then transferred to a nitrogen-free nutrient solution for 3 d. Subsequently, the plants were transplanted into 1 mM (^15^NH_4_)_2_SO_4_ with a ^15^N abundance of 5%. Samples were taken at 3, 6, and 24 h to determine the ^15^N content.

### Comparative transcriptome analysis

The seedlings were grown in a NO_3_^-^ nutrient solution for 5 d, and then transferred to a nitrogen-free nutrient solution for 3 d to deplete the stored NO_3_^-^. Subsequently, the plants were transferred to 1 mM Ca(NO_3_)_2_ or 1 mM (NH_4_)_2_SO_4_ for 1 d. The total RNA was then extracted from the roots of the NH_4_^+^-tolerant and -sensitive ecotypes. Three biological replicates were used for each ecotype under each treatment. The sequencing library was generated using the NEBNext Ultra™ RNA Library Prep Kit from Illumina (New York, NEB, USA), followed by sequencing on an Illumina HiSeq 2500 platform (San Diego, CA, USA). Gene expression levels were calculated using the FPKM method (Fragments Per Kilobase per Million mapped reads. Differentially expressed genes (DEGs) were defined as genes with |log_2_^(fold change)^ | >1 and a false discovery rate (FDR) < 0.05. The comparative transcriptome between NH_4_^+^ and NO_3_^-^ was identified through comparisons of the FPKM values for each gene between NH_4_^+^ and NO_3_^-^. The comparative transcriptome between the two genotypes was identified through comparisons of the FPKM values for each gene between Or-1 and the Rak-2. GO enrichment analysis was performed by the agriGo program (http://bioinfo.cau.edu.cn/agriGO/). The significantly enriched GO terms were defined with corrected *P* < 0.05.

### Statistical analysis

Graphical values represent the mean ± SD. Statistical analyses were conducted using two-tailed Student’s *t*-tests or one-way analysis of variance (ANOVA). Least significant difference (LSD) tests for different treatments were used for multiple comparisons with P < 0.05. The significance level was set at P < 0.05 (*) and *P* < 0.01 (**).

## Results

### The tissue NH_4_^+^ concentration was negatively linked to *Arabidopsis* growth

**Figure 1:**
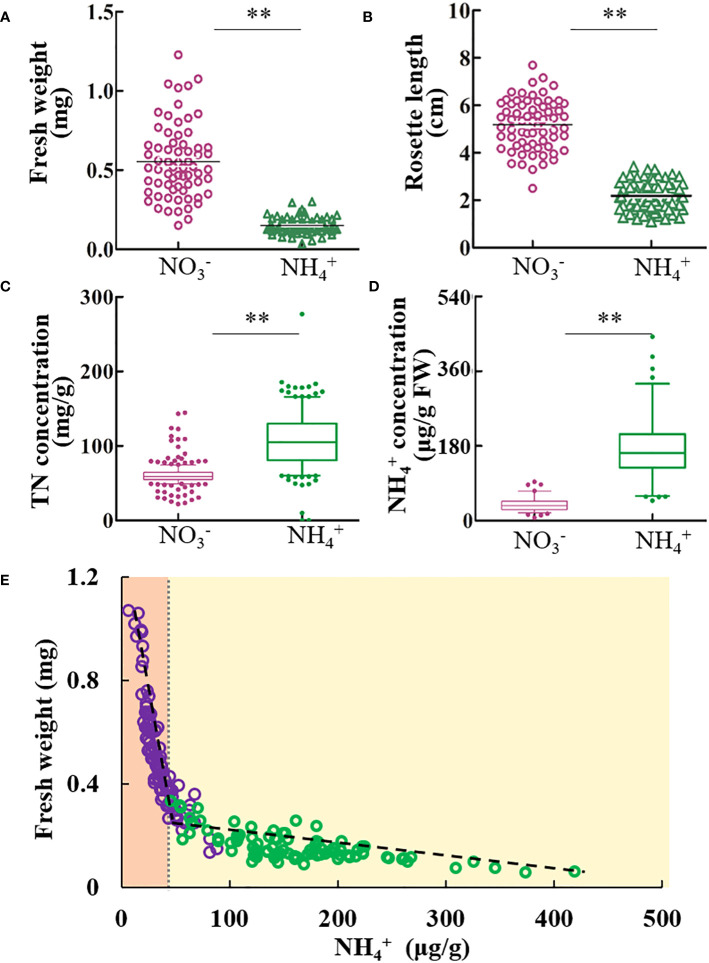
The tissue NH_4_^+^ concentration is negatively linked to the growth of Arabidopsis. After 8 d cultivation with NO_3_^-^ or NH_4_^+^ nutrition, the physiological parameters including the fresh weight, rosette length, nitrogen concentration and free NH_4_^+^ were investigated. **(A)** The fresh weight, and **(B)** rosette length under nitrate or NH_4_^+^ nutrition. **(C)** Total nitrogen concentration, and **(D)** free NH_4_^+^ concentration in the plants. **(E)** The correlation analysis of free NH_4_^+^ with fresh weight. Dashed lines indicated two linear models. Pearson R^2^ values are given when *p* <.05. Student’s t test (**p <.01) was used to analyze statistical significance. For fresh weight and rosette length, results of each ecotype are means of eight biological replicates, and for TN and free NH_4_^+^ concentration, results of each ecotype are means of three biological replicates.

A panel of *A. thaliana* natural accessions was grown under identical concentrations of NH_4_^+^ and NO_3_^-^. The growth of each ecotype was significantly inhibited by NH_4_^+^ (**Figures 1A, B**). Conversely, nitrogen concentration was significantly higher in plants grown under NH_4_^+^ than under NO_3_^-^ conditions (**Figure 1C**). However, the free NH_4_^+^ concentration in the shoots was significantly higher than that under NO_3_^-^, and its concentration varied widely among ecotypes (**Figure 1D**). The two-segment linear model accurately simulated the relationship between fresh weight and tissue NH_4_^+^ concentration under different nitrogen sources (**Figure 1E**, **Table S1**). The fresh weight sharply decreased with the tissue NH_4_^+^ concentration (<50 μg/g) under NO_3_^-^ conditions, and the pace of decline slowed when the NH_4_^+^ concentration was > 50 g/g under NH_4_^+^ conditions (**Figure 1E**). Our data revealed that the tissue NH_4_^+^ concentration was negatively linked to *Arabidopsis* growth, even under NO_3_^-^ culture conditions.

### Sensitivity to NH_4_^+^ between Or-1 and the Rak-2

**Figure 2:**
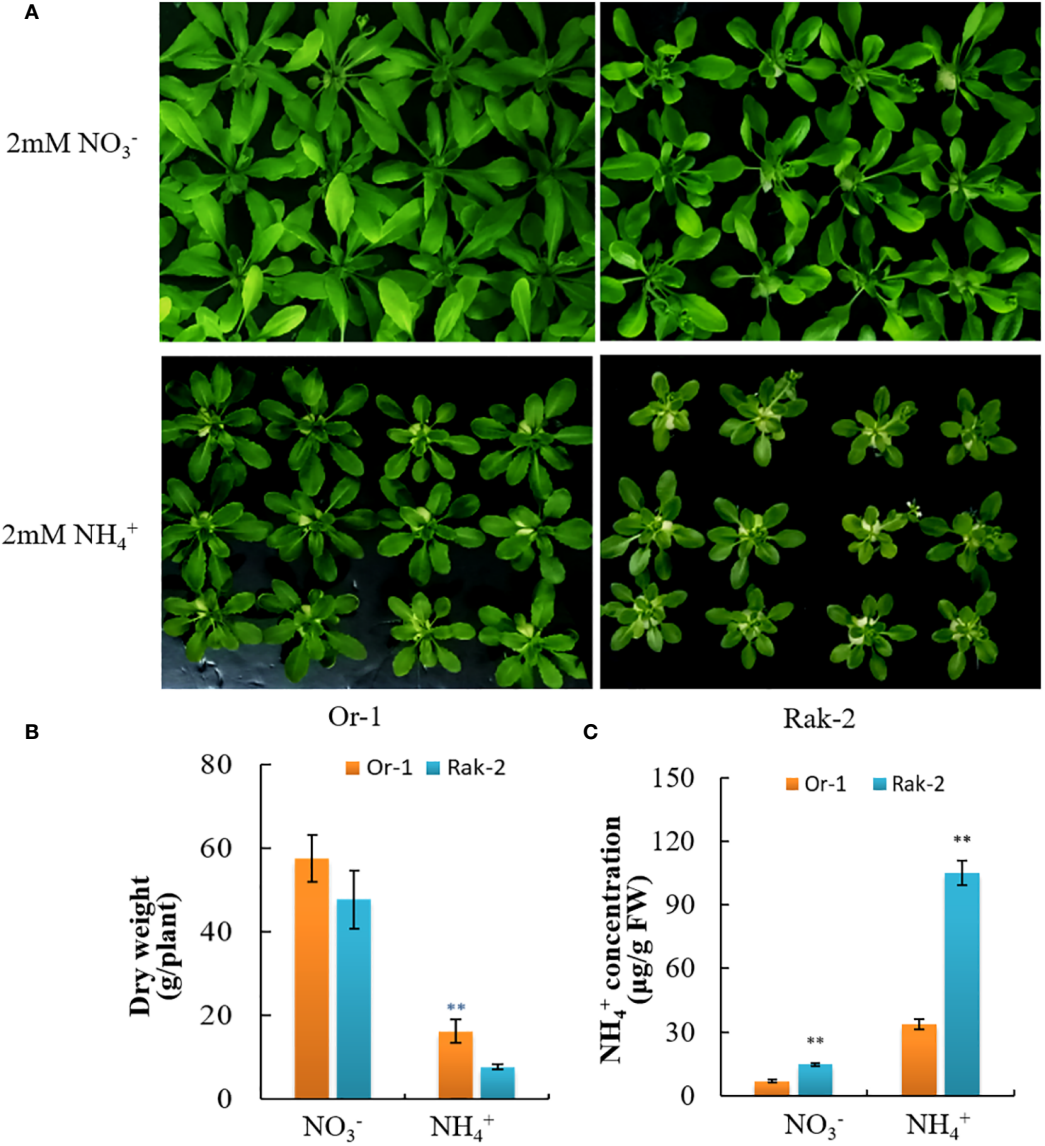
Different sensitivity to NH_4_^+^ nutrition between the Or-1 and the Rak-2. **(A)** Photos of the two ecotypes under NO_3_^-^ or NH_4_^+^ nutrition. **(B)** The dry weight of whole plants, and **(C)** free NH_4_^+^ concentration of the two ecotypes under NO_3_^-^ or NH_4_^+^ nutrition. For dry weight, results of each ecotype are means ± SD of eight biological replicates, and for free NH_4_^+^ concentration, results of each ecotype are means ± SD of three biological replicates. **p < .01.

We comprehensively considered the biomass under ammonium nitrogen and the biomass under normal conditions, and excluded the ecotypes with poor growth under nitrate nitrogen. Additionally, the Or-1 and Rak-2 showed similar growth period. Thus, ecotypes Or-1 and Rak-2 were selected for further investigation. Or-1 grew better under both NO_3_^-^ and NH_4_^+^ nutrition (**Figure 2A**), which was also supported by its biomass (**Figure 2B**). With NO_3_^-^ nutrition, the biomass of Rak-2 was 18.4% smaller than that of Or-1. However, the difference in biomass between the two ecotypes was 53% under NH_4_^+^ nutrition (**Figure 2B**). The free NH_4_^+^ concentration in Rak-2 was significantly higher than that in Or-1 under both nitrate and NH_4_^+^ nutrition conditions (**Figure 2C**). Thus, Or-1 was characterized as an NH_4_^+^-tolerant ecotype with high biomass and low NH_4_^+^ concentration, while Rak-2 was characterized as an NH_4_^+^ -sensitive ecotype.

The response of the transcriptome to NH_4_^+^ was analyzed to explore the underlying molecular mechanisms. In Or-1, 1016 genes were upregulated and 687 genes were downregulated by NH4+ (**Figure S1A**). In Rak-2 cells, 483 and 637 genes were upregulated and downregulated by NH4+, respectively (**Figure S1B**). NO_3_^–^responsive genes (e.g., *NRT2.1*, *NIA1*, *G6PD3*, *and FNR2*) were activated by NO_3_^-^ in both ecotypes (**Figure S1**). Based on the fold change and FDR of the differentially expressed genes (DEGs), the genes (*CLE4, PMEI4, FAR3, HHO1*) were strongly activated by NO_3_^-^, and the genes (*ALMT1, CCX1, ALD1, PBS3, DUR3*) were strongly activated by NH_4_^+^ (**Figure S1**).

### Core biological processes and pathways involved in the responses to NH_4_^+^

**Figure 3:**
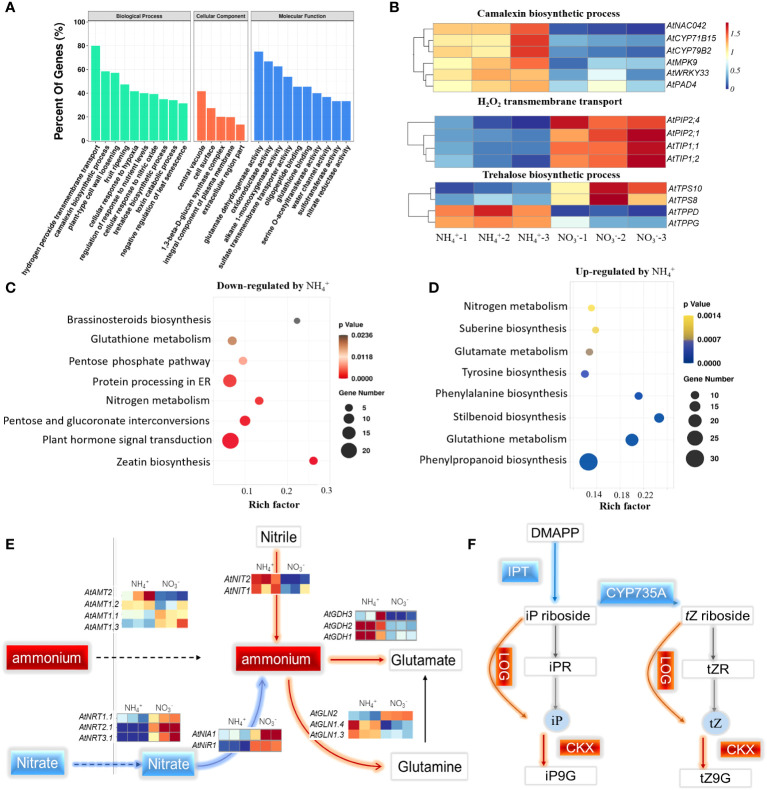
Overview of the transcriptome and the DEGs that were highly enriched in the core biological process and pathways in response to NH_4_^+^. **(A)** Gene ontology (GO) enrichment analysis of the DEGs under NH_4_^+^ stress. **(B)** The expression profiling of the genes involving in camalexin biosynthesis, H_2_O_2_ transmembrane transport and trehalose biosynthesis. **(C)** The enrichment pathways of the down-regulated genes by NH_4_^+^. **(D)** The enrichment pathways of the up-regulated genes by NH_4_^+^. **(E)** The effects of NH_4_^+^ nutrition on the pathway of nitrogen assimilation. **(F)** The effects of NH_4_^+^ nutrition on the pathway of zeatin biosynthesis. The red lines indicated the process was facilitated and the blue lines indicated the process was inhibited by NH_4_^+^.

The DEGs in the two ecotypes were used to analyze the core biological processes and pathways in response to NH_4_^+^. The Gene Ontology (GO) enrichment analysis indicated that genes involved in hydrogen peroxide transport, camalexin biosynthesis, trehalose biosynthesis, and glutamate dehydrogenase activity were the most enriched terms regulated by NH_4_^+^ (**Figure 3A**). The genes (*PIP2.1, PIP2.4, TIP1.1, TIP1.2*), annotated to transport H_2_O and H_2_O_2_, respectively, were repressed by NH_4_^+^ (**Figure 3B**). However, the genes (NAC042, *CYP71B15, AtCYP71B2, MPK9, WRKY33, PAD4)* that participate in camalexin biosynthesis, were activated by NH_4_^+^ (**Figure 3B**). The trehalose-6-phosphate synthesis genes (*TPS8, TPS 10*) were repressed by NH_4_^+^, while the trehalose-6-phosphate phosphatase genes (*TPPD, TPPG*) were activated (**Figure 3B**). The results suggest that NH_4_^+^ represses the process of water transport and trehalose-6-phosphate synthesis but induces camalexin synthesis.

By analyzing the up- or downregulated gene sets, the KEGG results showed that the most enriched pathways were activated or repressed by NH_4_^+^. The zeatin biosynthesis pathway was the most enriched process and was repressed by NH_4_^+^ (**Figure 3C**). The isopentenyl-transferase gene (IPT) and the cytochrome P450 monooxygenase CYP735A encodes the rate-limiting enzyme in CK synthesis. Surprisingly, the transcript levels of *IPT3, 5, 7* with NH_4_^+^ nutrients were 6%, 39%, and 22%, respectively, of those under NO_3_^-^ nutrients, and the transcript levels of *CYP735A1, 2* under NH_4_^+^ nutrients were only 10% and 15%, respectively, of those under NO_3_^-^ nutrient conditions (**Figure 3E**; **Figure S2**). However, the CK oxidase genes (*CKX1, 5, 6*) under NH_4_^+^ nutrient were 1.6 and 3.1 times higher than that under nitrate nutrient. These results support the conclusion that NH_4_^+^ represses CK synthesis but reduced the level of active CK.

The most enriched pathway activated by NH_4_^+^ was stilbenoid biosynthesis, followed by phenylalanine biosynthesis, glutathione metabolism, phenylpropanoid biosynthesis, and glutamate metabolism (**Figure 3D**). Nitrogen metabolism was simultaneously enriched in the upregulated and downregulated pathways, as the nitrate transporters (*NRT1.1, NRT2.1, NRT3.1*) and reduction genes (*NIA1, NiR1*) were strongly repressed by NH_4_^+^, and NH_4_^+^ assimilation genes (*GLN1.4, GLN1.3, GDH3, GDH2, GDH1*) were highly activated by NH_4_^+^ (**Figure 3E**).

### Stilbenoid, phenylpropanoid, and sucrose metabolism enriched in the tolerant ecotype were up-regulated by NH_4_^+^

**Figure 4:**
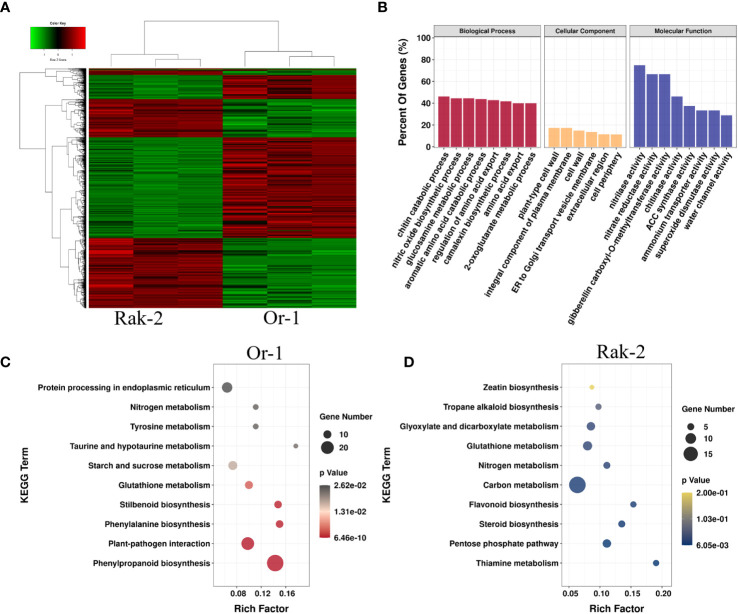
Transcriptional characterization of the DEGs between the NH_4_^+^-tolerant ecotype and the NH_4_^+^-sensitive ecotype. **(A)** The expression profiling of DGEs between the NH_4_^+^-tolerant ecotype and the NH_4_^+^-sensitive ecotype. **(B)** GO enrichment analysis of the DGEs. **(C)** The enrichment pathways of the DGEs with higher expression in the NH_4_^+^-tolerant ecotype Or-1. **(D)** The enrichment pathways of the DGEs with higher expression in the NH_4_^+^-sensitive ecotype Rak-2. The circle size indicates the number of DEGs, and the rich factor indicates the degree of enrichment of the KEGG pathways.

To explore the molecular mechanism of NH_4_^+^ sensitivity in Or-1 and Rak-2, we compared the transcriptomes of the NH_4_^+^-sensitive ecotype Rak-2 and the NH_4_^+^-tolerant ecotype Or-1, under NH_4_^+^ nutrient conditions. Overall, there were 2460 DEGs between the two ecotypes (**Figure 4A**; **Figure S3**). Among them, the expression of 1291 genes were higher in the NH_4_^+^-tolerant ecotype Or-1, which was more than the number of genes with higher expression in the NH_4_^+^-sensitive ecotype Rak-2 (**Figure 4A**). The functions of these DEGs were predicted using GO enrichment analysis. Some enriched GO terms between the two genotypes overlapped with those that responded to NH_4_^+^, such as camalexin biosynthesis, water channel activity, and nitrilase activity (**Figure 4B**; **Figure 3A**).

By analyzing the higher expression genes in the Rak-2 compared with the Or-1, The most enriched pathways were found to be thiamine metabolism, pentose phosphate pathway (**Figure 4D**). Analysis of genes with higher expression in Or-1 than in Rak-2 under NH_4_^+^-rich conditions. The most enriched pathways included stilbenoid, phenylpropanoid, glutathione, and sucrose metabolism (**Figure 4C**). Importantly, these pathways enriched in the Or-1 were up-regulated by NH_4_^+^ (**Figure S4**).

### The NH_4_^+^-tolerant ecotype Or-1 exhibits a more intensive response to NH_4_^+^ by activating defense processes and pathways

**Figure 5:**
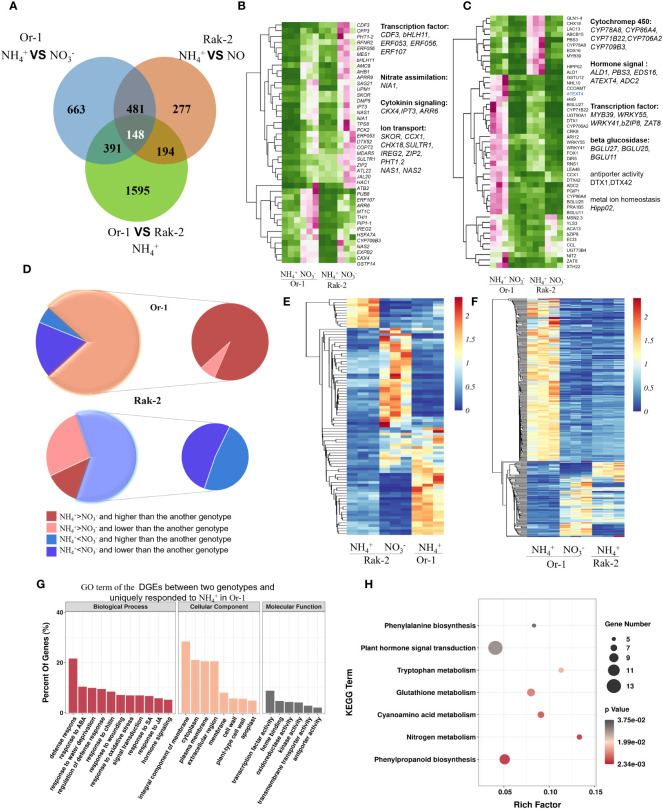
Analysis of the common and specific NH_4_^+^-responsive genes between the two ecotypes. **(A)** The VENN diagram of the DEGs. **(B)** The commonly down-regulated DEGs between the two ecotypes. **(C)** The commonly down-regulated DEGs between the two ecotypes. **(D)** The DEGs between the two ecotypes specifically responded to NH_4_^+^ only in one ecotype. **(E)** The expression profiling of the DEGs specifically responded to NH_4_^+^ in the sensitive ecotype Rak-2. **(F)** The expression profiling of the DEGs specifically responded to NH_4_^+^ in the tolerant ecotype Or-1. **(G)** GO enrichment analysis of the DGEs specifically responded to NH_4_^+^ in the tolerant ecotype Or-1. **(H)** The enrichment pathways of the DGEs specifically responded to NH_4_^+^ in the tolerant ecotype Or-1.

As shown in the VENN diagram, 148 DEGs between the two ecotypes were simultaneously responsive to NH_4_^+^ (**Figure 5A**). In addition, 391 DEGs between the two ecotypes specifically responded to NH_4_^+^ in the tolerant ecotype Or-1 (**Figure 5A**). Similarly, 194 DEGs between the two ecotypes specifically responded to NH_4_^+^ in the sensitive ecotype Rak-2 (**Figure 5A**). The core 148 genes were classified into upregulated and downregulated genes following NH_4_^+^ treatment (**Figures 5B, C**; **Table S2**). Among the core 148 genes, a higher proportion of genes were upregulated by NH_4_^+^, especially Or-1 (**Figure 5C**). The *cytochrome P450* genes *CYP86A4, CYP71B22, CYP706A2*, and *CYP709B3* were strongly induced by NH_4_^+^ and showed higher Or-1 expression (**Figure 5C**). The transcription factors *WRKY55, WRKY41, bZIP8*, and *ZAT8* were more strongly induced in the NH_4_^+^-tolerant ecotype Or-1 than that in the NH_4_^+^-sensitive ecotype Rak-2 (**Figure 5C**).

Surprisingly, 194 DEGs between the two ecotypes that specifically responded to NH_4_^+^ in Rak-2 were mostly downregulated by NH_4_^+^ (**Figures 5D, E**; **Table S2**). Conversely, 391 DEGs between the two ecotypes specifically responded to NH_4_^+^ in Or-1 plants (**Figures 5D, F**; **Table S2**). These genes were mostly upregulated by NH_4_^+^ and showed higher expression levels in Or-1 cells (**Figures 5D, F**). These results suggest that a large number of genes were uniquely upregulated in the NH_4_^+^- tolerant ecotype Or-1. The function of these unique NH_4_^+^-responsive genes in the Or-1 were predicted using GO enrichment analysis and KEGG pathways. The most highly enriched biological processes were defense responses, including responses to ABA, water deprivation, chitin, wounding, and oxidative stress (**Figure 5G**). Similarly, the KEGG pathway analysis indicated that nitrogen metabolism, phenylpropanoid biosynthesis, glutathione metabolism, and plant hormone signal transduction were the most highly enriched pathways (**Figure 5H**). Collectively, these results suggest that the NH_4_^+^-tolerant ecotype Or-1 exhibits a more intensive response to NH_4_^+^ by activating defense processes and pathways, including phenylpropanoid biosynthesis, nitrogen metabolism, and glutathione metabolism.

### The tolerant ecotype maintained a low NH_4_^+^ level, mainly by promoting NH_4_^+^ assimilation rather than inhibiting NH_4_^+^ uptake

**Figure 6:**
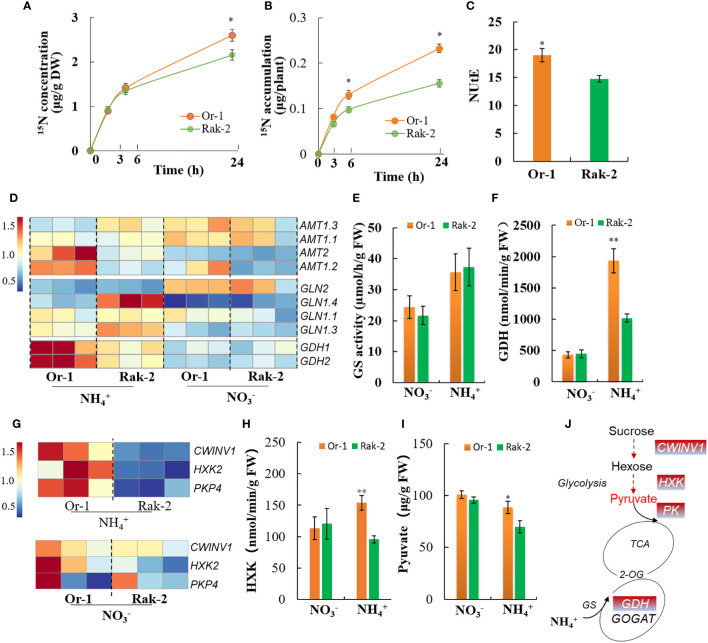
Analysis of NH_4_^+^ assimilation capacity between the two varieties. **(A, B)** The short-term of ^15^NH_4_^+^ uptake for 3 h, 6 h and 24 h. **(C)** The nitrogen utilization efficiency (NUtE) between the two ecotypes under NH_4_^+^ nutrition. NUtE is calculated by biomass/nitrogen. **(D)** Differential expression profiling of the NH_4_^+^ uptake and assimilation genes, including the NH_4_^+^ transporter genes *AMTs*, glutamine synthesis genes *GLN* and glutamate dehydrogenase genes *GDH*. **(E)** The activity of glutamine synthesis in the roots of two ecotypes. **(F)** The activity of GDH in the roots of two ecotypes. **(G)** Differential expression profiling of the carbohydrate metabolism genes, including the cell wall-bound invertase gene *CWINV1*, hexokinase gene *HXK2* and pyruvate kinase gene *PK4*. **(H)** The activity of hexokinase in the roots of two ecotypes. **(I)** The content of pyruvate in the roots of two ecotypes. **(J)** The schematic diagram of carbon and nitrogen metabolism. The enzymes and metabolite highlighted in red font represent higher activity or content in the root of tolerant ecotype. Results are means ± SD of three biological replicates. *p < .05 and **p < .01.

As the NH_4_^+^-sensitive ecotype, Rak-2 accumulated more NH_4_^+^ than the NH_4_^+^-tolerant ecotype Or-1 (**Figure 2C**), their uptake and assimilation of NH_4_^+^ were investigated. Both the concentration and accumulation of ^15^N were significantly higher in the NH_4_^+^-tolerant ecotype Or-1 (**Figures 6A, B**). The higher nitrogen utilization efficiency (NUtE) in the tolerant ecotype indicated the higher efficiency of the conversion of shoot N into shoot biomass (**Figure 6C**). The expression of *AMT2* and *AMT1.2* were also higher in the NH_4_^+^-tolerant ecotype (**Figure 6D**), which supports the results for ^15^NH_4_^+^ assimilation. Although the GS activity of both ecotypes increased with increasing NH_4_^+^ concentration, no difference was observed between the two (**Figure 6E**). The expression of *Gln1s* was significantly induced by NH_4_^+^, and only the expression of *Gln1.4* slightly higher in the NH_4_^+^ -sensitive ecotype (**Figure 6D**). However, GDH activity was strongly induced by NH_4_^+^ nutrition and was significantly higher in the tolerant ecotype when compared with that in the sensitive ecotype (**Figure 6F**). Accordingly, the expression of *GDH1* and *GDH2* was more significantly induced in the roots of the tolerant ecotype than in those of the sensitive ecotype (**Figure 6D**).

The vital nodes of C and N metabolism were also investigated. The expression of hexokinase gene (*HXK2*) and pyruvate kinase gene (*PK4*) were much higher in the roots of the tolerant ecotype than the sensitive ecotype with NH_4_^+^ nutrition (**Figure 6G**). These results are consistent with the enzymatic activities of HXK and PK (**Figure 6H**; **Figure S5**). Importantly, pyruvate content, which is essential for N metabolism, was reduced by NH_4_^+^ nutrition, whereas its content was significantly higher in the roots of the tolerant ecotype than in the sensitive ecotype under NH_4_^+^ nutrition (**Figure 6I**). These results indicated that the tolerant ecotype maintained a low NH_4_^+^ level, mainly by promoting NH_4_^+^ assimilation rather than inhibiting NH_4_^+^ uptake.

## Discussion

### The tissue NH_4_^+^ concentration is an important factor contributing to variations in plant growth and NH_4_^+^ tolerance

NH_4_^+^ is toxic to plants, even at micromolar concentrations (von Wirén et al., 2000). Many hypotheses have been proposed to explain NH_4_^+^ toxicity, such as the depletion of organic acids, deficiency of cations, futile transmembrane NH_4_^+^ cycling and so on (Li et al., 2014). Previous studies have explored the medium NH_4_^+^ concentration toxic to different crops (Britto and Kronzucker, 2002). A previous study has expounded the critical role of NH_4_^+^ in *Arabidopsis* natural variability in NH_4_^+^ tolerance (Sarasketa et al., 2014). In this study, we found the two-segment linear model accurately simulated the relationship between fresh weight and tissue NH_4_^+^ concentration under different nitrogen sources (**Figure 1E**). When the tissue NH_4_^+^ concentration was below 50 μg/g under nitrate conditions, the fresh weight rapidly decreased with the tissue NH_4_^+^ concentration. Whereafter, the pace of declines slowed, when the tissue NH_4_^+^ concentration was over 50 μg/g under NH_4_^+^ conditions, (**Figure 1E**). This phenomenon possibly enlightened that low concentrations of NH_4_^+^ do not cause obvious symptoms of NH_4_^+^ toxicity, such as leaf chlorosis, but affect plant growth in an imperceptible way. However, the visible symptoms of NH_4_^+^ toxicity appeared over 50 μg/g under NH_4_^+^ conditions. A previous study revealed that the shoot K^+^ was positively correlated with the growth and NH_4_^+^ tolerance (Chen et al., 2021), and this correlation may be indirectly caused by the inhibition of K^+^ transport. Thus, the tissue NH_4_^+^ concentration is an important factor contributing to variations in plant growth and NH_4_^+^ tolerance.

Plants deal with NH_4_^+^ overload by using different strategies, including NH_4_^+^ transporters and NH_4_^+^ assimilation regulation. Inactivation of AMT1;1 and AMT1;2 by interaction with CIPK23 is important for NH_4_^+^ uptake regulation; Thus, the Loss of CIPK23 increases root NH_4_^+^ uptake and confers hypersensitivity to NH_4_^+^ (Straub et al., 2017). In the present study, the NH_4_^+^-tolerant ecotype Or-1 accumulated more ^15^N-labeled NH_4_^+^ (**Figures 6A, B**), thus the repression of AMT transporter cannot explain the tolerance mechanism of Or-1. Instead, the NH_4_^+^-tolerant ecotype displayed higher expression of *AMT2* and *AMT1.2* than the NH_4_^+^ -sensitive ecotype Rak-2, and its expression were induced by NH_4_^+^ (**Figure 6D**). Recently study found the expression of *ZmAMT1s* were induced by NH_4_^+^ nutrition and discovered glutamine rather than NH_4_^+^ regulated ZmAMT1s (Hui et al., 2022). The higher content of assimilated ^15^N-labeled H_4_^+^ and NUtE indicated that the tolerant ecotype Or-1 had a stronger NH_4_^+^ assimilation capacity, thus induced the expression of *AMTs* through a positive feedback by the NH_4_^+^ assimilate - glutamine.

### The tolerant ecotype had a stronger NH_4_^+^ assimilation capacity to alleviating NH_4_^+^ toxicity

In plants, NH_4_^+^ assimilation generally occurs via the GS/GOGAT cycle. Cytosolic (GS1) and chloroplast (GS2) isoforms play opposing roles in NH_4_^+^ stress. Root Gln1.2 alleviates NH_4_^+^ toxicity, whereas shoot Gln2 aggravates NH_4_^+^ toxicity in a pH-dependent manner (Hachiya et al., 2021). Indeed, the expression of *Gln1s* was significantly induced by NH_4_^+^, whereas the expression of *Gln2* was repressed (**Figure 6D**), suggesting an interesting regulation of *GS* to adapt NH_4_^+^ stress in plants. Although the GS activity of both ecotypes increased with increasing NH_4_^+^ concentration, no difference was observed between the two ecotypes (**Figure 6E**). Another study found no correlation between GS activity and shoot biomass in *Arabidopsis* under NH_4_^+^ nutrition (Sarasketa et al., 2014). Thus, variations in GS activity may not be crucial for NH_4_^+^ tolerance in different Arabidopsis ecotypes. GDH can incorporate NH_4_^+^ independently of the GS/GOGAT cycle and plays a critical role in the detoxification of NH_4_^+^ in stress conditions (Xian et al., 2020). Although the capacity of GDH to synthesize Glu *in vivo* has not been clearly demonstrated, heterologous expression of fungal GDH in plants could alleviates NH_4_^+^ toxicity and improve nitrogen assimilation (Tang et al., 2018; Yan et al., 2021). In this study, GDH activity and its encoding genes in both ecotypes were induced by NH_4_^+^ nutrition, and the activity was much higher in the tolerant ecotype than in the sensitive ecotype (**Figure 6F**). Moreover, *GDH1* and *GDH2* were more strongly induced in the roots of the tolerant ecotype than in those of the sensitive ecotype (**Figure 6D**). Overall, these results possibly indicate the critical role of GDH in *Arabidopsis* natural variation in NH_4_^+^ tolerance.

The sucrose metabolism pathway was enriched in the tolerant ecotype, indicating that this process was more active in the NH_4_^+^-tolerant ecotype (**Figure 4C**; **Figure S4**). Hexokinase (HXK) and Pyruvate kinase (PK) function crucial roles in Glycolysis and TCA cycle, providing 2-OG and energy for N metabolism (Stitt, 1999). In the present work, the expression of *HXK2* and *PK4* were much higher in the root of tolerant ecotype than the sensitive ecotype under NH_4_^+^ nutrition (**Figure 6G**). These results are consistent with the enzymatic activities of HXK and PK (**Figure 6H**; **Figure S5**). Importantly, the crucial carbon metabolite pyruvate was reduced by NH_4_^+^ nutrition, whereas its content was significantly higher in the roots of the tolerant ecotype than in the sensitive ecotype under NH_4_^+^ nutrition (**Figure 6I**). These results suggest that the tolerant ecotype had a stronger carbon skeleton (2-OG) production capacity for NH_4_^+^ assimilation (**Figure 6J**).

Coordination of carbon (C) and nitrogen (N) metabolism is essential for plant growth and stress tolerance (Reguera et al., 2013; Liang et al., 2020). The higher nitrogen utilization efficiency (NUtE) in the tolerant ecotype indicated the higher efficiency of the conversion of shoot N into shoot biomass (**Figure 6C**). The NUtE was similar among the accessions with different nitrogen use efficiency and uniformly decreased with high N supply, but the accessions differed in their NUtE under N restriction (Menz et al., 2018). The nitrogen was not limited and the concentration was even higher under NH_4_^+^ nutrition than that under nitrate nutrition. Thus, the higher NUtE in the tolerant ecotype may suggest better-coordinated C/N metabolism compared with the sensitive ecotype under NH_4_^+^ stress.

### The tolerant ecotype displayed stronger defense responses by activating phenylpropanoids and the derived stilbenoids

NH_4_^+^ induces ROS formation, leading to oxidative damage in plants (Podgorska et al., 2013; Liu et al., 2022). Here, stilbenoid biosynthesis, phenylpropanoid biosynthesis, and glutathione metabolism pathways were commonly activated by NH_4_^+^ in all ecotypes (**Figure 3D**). Stilbenoids, which are hydroxylated derivatives of stilbene belonging to the phenylpropanoid family, have been shown to have a wide spectrum of biological functions, such as antioxidant and antimicrobial activities (Dong and Lin, 2021). Many other secondary metabolites, such as anthocyanins and flavonols, share a common origin in the phenylpropanoid biosynthetic pathway and functions as an ROS scavenger induced by abiotic stress (Dong and Lin, 2021). Treatment with flavonoids, such as naringenin, reduces oxidative damage under Cd stress in rice (Chen et al., 2022). Moreover, phenylpropanoid metabolism is associated with the reinforcement of cell walls under NH_4_^+^ nutrition, which was considered as an important NH_4_^+^ tolerance mechanism (Royo et al., 2019; Yang et al., 2022). Importantly, the NH_4_^+^-tolerant ecotype Or-1 showed a more intense response to NH_4_^+^ by activating pathways, including phenylpropanoid and stilbenoid biosynthesis (**Figure 4C**). Therefore, the biosynthesis of phenylpropanoids and stilbenoids derived from NH_4_^+^ may play a positive role in the defense response to NH_4_^+^ toxicity, and this thus warrants further investigation.

The tolerant ecotype was more responsive to NH_4_^+^ stress than the sensitive ecotype. These genes were highly enriched in defense responses, including their responses to ABA, water deprivation, chitin, wounding, and oxidative stress (**Figure 5G**). ABA signaling plays a key role in oxidative stress responses and is associated with the induction of antioxidant defense systems (Yang et al., 2015; Li et al., 2019b). Interestingly, the NH_4_^+^- tolerant ecotype has stronger ABA responses and phenylpropanoid metabolism for ROS scavenging (**Figures 5G, H**). Recently, a research investigated the genetic variation underlying differential ammonium and nitrate responses in *Arabidopsis thaliana*, and found the preferring ammonium or nitrate, appeared to be generated by combinations of loci rather than a few large-effect loci, which most are specific to a developmental or defense trait under specific nitrogen source (Katz et al., 2022). In our results, we also found only few genes were simultaneously responsive to NH_4_^+^ between the two genotypes (**Figure 5A**). This suggested that ammonium tolerance genotypes are a combination of multiple mini-effect genes.

## Conclusion

In this study, the tissue content of NH_4_^+^ was found the main cause for NH_4_^+^ toxicity, and the two-segment linear model accurately simulated the relationship between fresh weight and tissue NH_4_^+^ concentration under different nitrogen sources. we revealed that the tolerant ecotype maintained a low NH_4_^+^ level, mainly by promoting NH_4_^+^ assimilation rather than inhibiting NH_4_^+^ uptake. The carbon and nitrogen metabolism analysis revealed that the tolerant ecotype had a stronger carbon skeleton (2-OG) production capacity with higher levels of hexokinase (HXK), pyruvate kinase (PK), and GDH activity to assimilate free NH_4_^+^. Furthermore, the core information about the biochemical regulation of plants in response to NH_4_^+^ toxicity was identified. The most enriched pathways included nitrogen metabolism, camalexin, stilbenoid and phenylpropanoid biosynthesis were upregulated by NH_4_^+^. Interestingly, a large number of genes, which enriched in phenylpropanoid and stilbenoid biosynthesis, were uniquely upregulated in the NH_4_^+^- tolerant ecotype. These results suggested that the NH_4_^+^-tolerant ecotype showed a more intense response to NH_4_^+^ by activating defense processes and pathways.

## Data availability statement

The datasets presented in this study can be found in online repositories. The names of the repository/repositories and accession number(s) can be found below: Gene Expression Omnibus, GSE243624.

## Author contributions

HC: Conceptualization, Data curation, Formal analysis, Writing – original draft. WL: Data curation, Formal analysis, Methodology, Writing – original draft. WZ: Investigation, Software, Validation, Writing – review & editing. JZ: Data curation, Formal analysis, Project administration, Writing – review & editing. QZ: Writing – review & editing. ZZ: Resources, Supervision, Writing – review & editing.
