# Supplementary material for: Integrated comparative transcriptome and physiological analysis reveals the metabolic responses underlying genotype variations in NH4 + tolerance
Source: Front Plant Sci. 2023 Dec 13;14:1286174. doi: 10.3389/fpls.2023.1286174 (PMC10773859; doi:10.3389/fpls.2023.1286174)
Supplement: Supplementary file 1 [file DataSheet_1.docx]

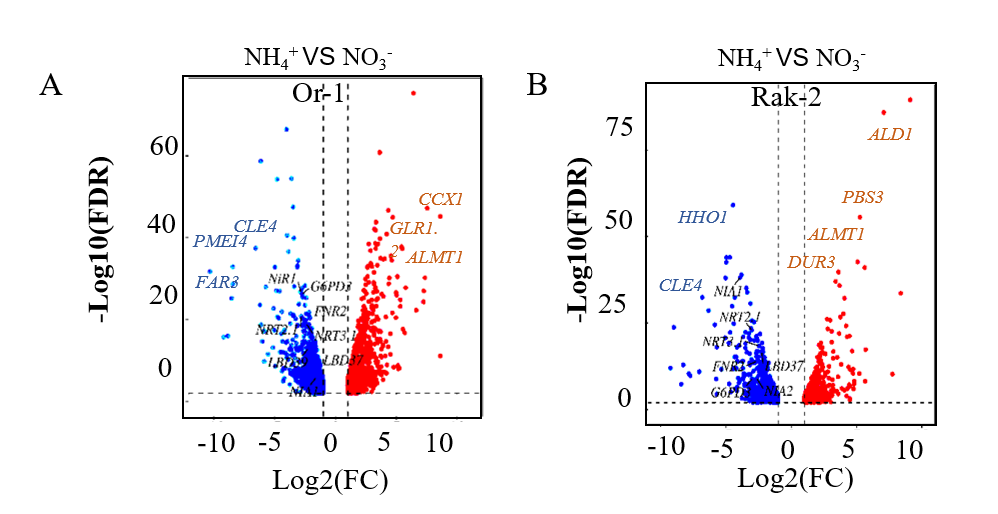


**Supplementary Figure S1** The differentially expressed genes (DGEs) were identified through comparisons of the FPKM values for each gene between ammonium and nitrate (NH_4_^+^ vs NO_3_^-^) in the Or-1 (A). The DGEs were identified through comparisons of the FPKM values for each gene between ammonium and nitrate (NH_4_^+^ vs NO_3_^-^) in the Rak-2 (B). The DEGs were defined as genes with the FDR < 0.05 and with |log_2_ ^(fold change)^ | >1. The general nitrate-responsive genes (eg., *NRT2.1*, *NIA1*, *G6PD3*, *FNR2*) were marked with red as quality control index. The most outstanding up-regulated genes were marked with crown and the most outstanding down-regulated genes were marked with blue.


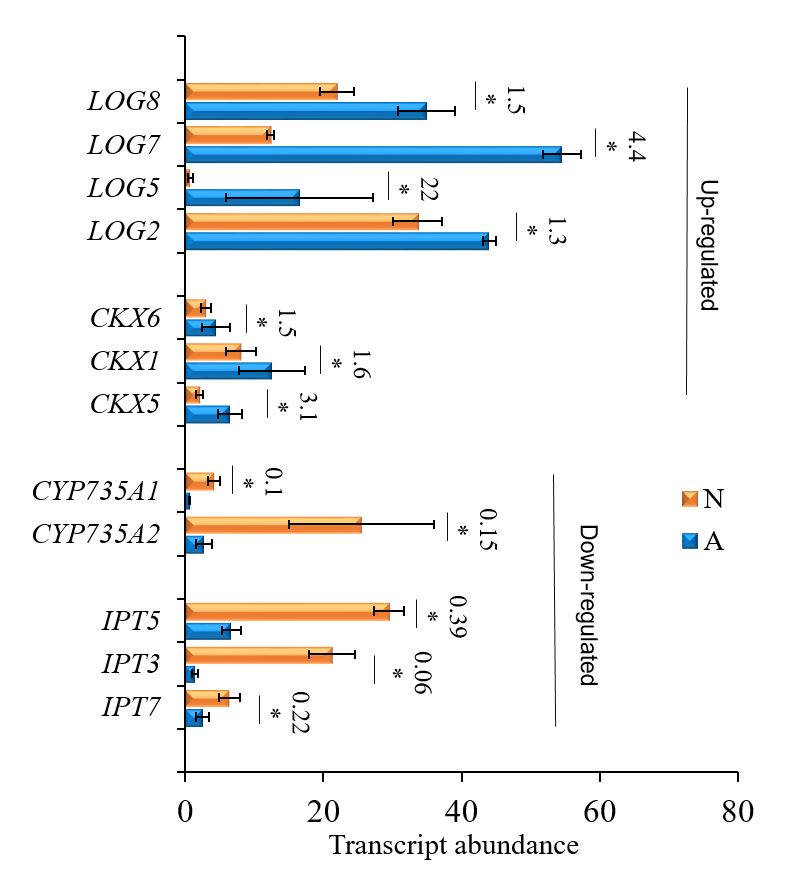


**Supplementary Figure S2** The effects of ammonium nutrition on the pathway of zeatin biosynthesis. The yellow bar represents nitrate-based nitrogen nutrition conditions (N). The blue bar represents the ammonium-based nitrogen nutrition conditions (A).


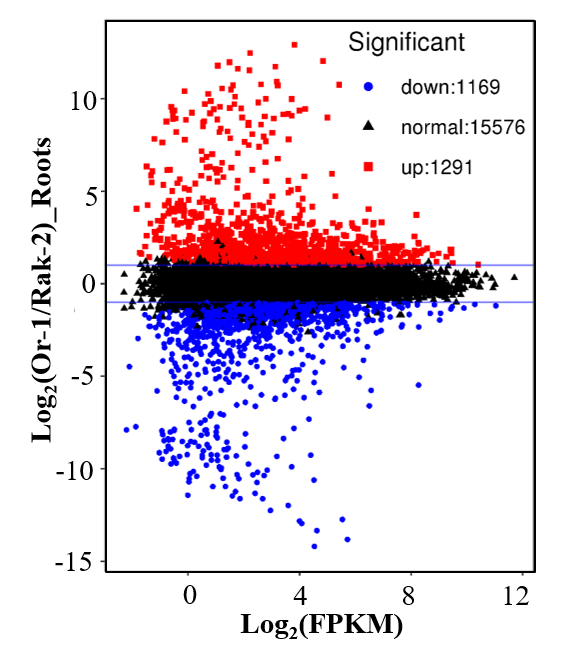


**Supplementary Figure S3** The overall DEGs between the two ecotypes.


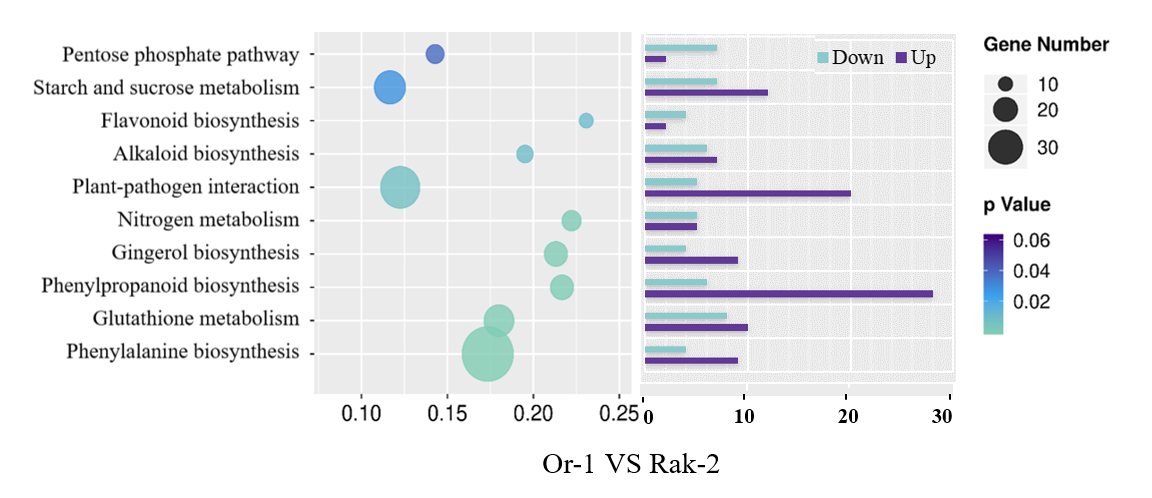


**Supplementary Figure S4** GO enrichment analysis of the DGEs between the two genotypes.


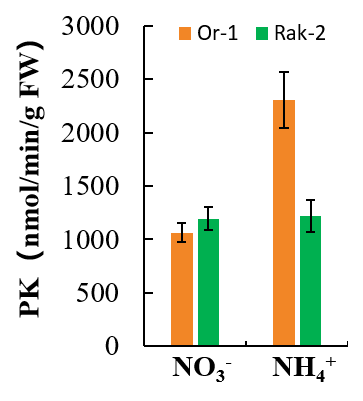


**Supplementary Figure S5** the enzyme activity of pyruvate kinase (PK) in the roots of two ecotypes.

**Supplementary Table 1** The traits of fresh weight and free NH_4_^+^ among the natural accessions of *Arabidopsis thaliana*

| Ecotypes | Ca(NO_3_)_2_ | |  | (NH4)_2_SO_4_ | |
| --- | --- | --- | --- | --- | --- |
|  | NH_4_^+^ (μg/g FW) | FW (mg) |  | NH_4_^+^ (μg/g FW) | FW (mg) |
| Gic-0 | 82.06 | 0.14 |  | 373.42 | 0.06 |
| ukID48 | 88.57 | 0.15 |  | 418.88 | 0.06 |
| Di-1 | 81.94 | 0.19 |  | 345.67 | 0.08 |
| 1458-A9 | 56.19 | 0.22 |  | 309.04 | 0.08 |
| Hi-0 | 67.66 | 0.25 |  | 168.45 | 0.09 |
| MZ-0 | 58.65 | 0.25 |  | 145.19 | 0.10 |
| Es5-1 | 64.94 | 0.25 |  | 325.89 | 0.10 |
| Tscha-1 | 59.50 | 0.25 |  | 120.53 | 0.10 |
| ga-2 | 50.73 | 0.26 |  | 264.41 | 0.10 |
| Lpv-18 | 44.02 | 0.27 |  | 160.25 | 0.11 |
| LM-2 | 50.32 | 0.28 |  | 200.32 | 0.11 |
| fi-1 | 50.95 | 0.28 |  | 259.85 | 0.11 |
| 2P2-6 | 63.07 | 0.30 |  | 247.55 | 0.11 |
| 917-D4 | 50.15 | 0.30 |  | 261.08 | 0.11 |
| Hey-1 | 48.74 | 0.30 |  | 141.88 | 0.11 |
| udull-34 | 48.30 | 0.31 |  | 210.77 | 0.12 |
| 10-2 | 52.88 | 0.32 |  | 125.05 | 0.12 |
| Jm-1 | 42.25 | 0.32 |  | 161.84 | 0.12 |
| DraIv1-f | 63.53 | 0.32 |  | 161.89 | 0.12 |
| CS28181 | 43.44 | 0.32 |  | 245.71 | 0.12 |
| Poy-0 | 42.67 | 0.32 |  | 267.55 | 0.12 |
| Ge-1 | 44.85 | 0.33 |  | 160.06 | 0.12 |
| uk-2 | 36.89 | 0.34 |  | 160.55 | 0.12 |
| Tou-A1-6f | 38.78 | 0.35 |  | 217.03 | 0.12 |
| Li-7 | 46.75 | 0.35 |  | 202.30 | 0.13 |
| wc-2 | 67.50 | 0.36 |  | 210.12 | 0.13 |
| kc-5 | 46.63 | 0.38 |  | 122.47 | 0.13 |
| D0-0 | 45.75 | 0.38 |  | 180.87 | 0.13 |
| 1277-1-1-7 | 38.15 | 0.38 |  | 142.23 | 0.13 |
| Pro-0 | 36.38 | 0.38 |  | 142.23 | 0.13 |
| Ha-0 | 42.63 | 0.38 |  | 143.67 | 0.13 |
| Krot-2 | 44.50 | 0.39 |  | 172.30 | 0.13 |
| Ste-0 | 40.97 | 0.39 |  | 181.73 | 0.13 |
| ZDH-6 | 52.45 | 0.40 |  | 184.79 | 0.13 |
| Lt-1 | 30.44 | 0.41 |  | 175.70 | 0.13 |
| Ba-1 | 34.44 | 0.41 |  | 222.94 | 0.13 |
| Lnh-0 | 40.32 | 0.42 |  | 143.67 | 0.13 |
| Nh-1 | 34.91 | 0.42 |  | 146.91 | 0.13 |
| NOK-1 | 31.11 | 0.42 |  | 154.53 | 0.13 |
| zw-1 | 30.77 | 0.42 |  | 188.29 | 0.13 |
| No-0 | 34.24 | 0.42 |  | 196.96 | 0.14 |
| Sh-0 | 44.48 | 0.43 |  | 223.62 | 0.14 |
| Bsch-0 | 36.56 | 0.43 |  | 124.79 | 0.14 |
| Ler-1 | 38.66 | 0.45 |  | 134.81 | 0.14 |
| Ph2-23 | 35.70 | 0.45 |  | 134.76 | 0.14 |
| Edrl2-2y | 33.85 | 0.46 |  | 101.76 | 0.14 |
| Ba-0 | 36.75 | 0.47 |  | 193.82 | 0.14 |
| nod-7 | 32.30 | 0.47 |  | 123.95 | 0.15 |
| Bay-0 | 36.93 | 0.47 |  | 202.47 | 0.15 |
| Wt-5 | 32.64 | 0.48 |  | 150.09 | 0.15 |
| Ba-0 | 34.27 | 0.50 |  | 174.93 | 0.15 |
| Rak-2 | 29.92 | 0.50 |  | 217.90 | 0.15 |
| MIB-22 | 28.39 | 0.50 |  | 185.16 | 0.15 |
| EP-0 | 32.32 | 0.51 |  | 179.11 | 0.15 |
| Ts-1 | 36.56 | 0.51 |  | 195.67 | 0.15 |
| Bur-0 | 31.28 | 0.51 |  | 197.25 | 0.15 |
| Mnz-0 | 28.17 | 0.52 |  | 121.74 | 0.16 |
| 1187-G7 | 30.98 | 0.53 |  | 139.63 | 0.16 |
| Nd-1 | 25.78 | 0.53 |  | 222.29 | 0.16 |
| Es-0 | 25.01 | 0.53 |  | 225.28 | 0.16 |
| MZB-15 | 27.88 | 0.53 |  | 179.17 | 0.17 |
| 8-DISte-3 | 35.53 | 0.54 |  | 125.46 | 0.17 |
| Rmx-A180 | 30.43 | 0.54 |  | 100.80 | 0.18 |
| Alst-1 | 26.08 | 0.56 |  | 181.75 | 0.18 |
| kl-5 | 24.50 | 0.57 |  | 106.49 | 0.18 |
| Paw-3 | 22.95 | 0.58 |  | 89.33 | 0.18 |
| CSHL-5 | 28.02 | 0.60 |  | 180.04 | 0.18 |
| Ang-0 | 26.47 | 0.60 |  | 203.54 | 0.18 |
| Hovdala-2 | 31.38 | 0.61 |  | 143.85 | 0.19 |
| Sei-0 | 31.70 | 0.61 |  | 56.64 | 0.19 |
| Ka-0 | 25.16 | 0.61 |  | 153.83 | 0.19 |
| Gr-1 | 23.95 | 0.62 |  | 107.28 | 0.19 |
| fab-4 | 34.03 | 0.62 |  | 89.17 | 0.19 |
| Bor-4 | 22.10 | 0.62 |  | 141.96 | 0.19 |
| Kyo-0 | 24.72 | 0.63 |  | 120.66 | 0.19 |
| Ko-0 | 20.88 | 0.64 |  | 107.28 | 0.19 |
| Rsch-4 | 26.60 | 0.64 |  | 136.86 | 0.20 |
| L7r-5 | 25.16 | 0.65 |  | 107.14 | 0.20 |
| In-w | 27.17 | 0.67 |  | 109.49 | 0.20 |
| Bx2 | 23.63 | 0.67 |  | 63.21 | 0.21 |
| Ha-0 | 22.12 | 0.68 |  | 149.58 | 0.21 |
| Bay-0 | 25.33 | 0.68 |  | 151.64 | 0.21 |
| Reh-1 | 23.12 | 0.70 |  | 140.35 | 0.22 |
| mt-0 | 23.17 | 0.71 |  | 79.27 | 0.22 |
| Mzk-0 | 25.67 | 0.74 |  | 120.32 | 0.24 |
| Fr-4 | 18.95 | 0.75 |  | 180.03 | 0.24 |
| An-2 | 23.97 | 0.76 |  | 64.82 | 0.25 |
| GD-1 | 18.82 | 0.86 |  | 104.04 | 0.26 |
| Tor-1 | 19.40 | 0.88 |  | 72.93 | 0.26 |
| Si-0 | 19.96 | 0.94 |  | 161.33 | 0.26 |
| Kno-18 | 13.77 | 0.97 |  | 63.24 | 0.27 |
| NW-0 | 19.04 | 0.99 |  | 70.54 | 0.31 |
| HSM | 18.07 | 1.00 |  | 54.57 | 0.31 |
| Or-1 | 12.64 | 1.02 |  | 54.57 | 0.31 |
| Tor-3 | 15.93 | 1.06 |  | 54.21 | 0.32 |
| Ta-0 | 6.67 | 1.07 |  | 45.71 | 0.34 |
